# Mechanical Circulatory Support of the Critically Ill Child Awaiting Heart Transplantation

**DOI:** 10.2174/157340310790231617

**Published:** 2010-02

**Authors:** Avihu Z Gazit, Sanjiv K Gandhi, Charles C Canter

**Affiliations:** 1 Division of Pediatric Critical Care, Saint Louis Children’s Hospital, Washington University School of Medicine, St Louis, Mo., USA; 2 Division of Pediatric Cardiothoracic Surgery, Saint Louis Children’s Hospital, Washington University School of Medicine, St Louis, Mo., USA; 3 Division of Pediatric Cardiology, Saint Louis Children’s Hospital, Washington University School of Medicine, St Louis, Mo., USA

**Keywords:** Ventricular assist device, heart failure, heart transplantation.

## Abstract

The majority of children awaiting heart transplantation require inotropic support, mechanical ventilation, and/or extracorporeal membrane oxygenation (ECMO) support. Unfortunately, due to the limited pool of organs, many of these children do not survive to transplant. Mechanical circulatory support of the failing heart in pediatrics is a new and rapidly developing field world-wide. It is utilized in children with acute congestive heart failure associated with congenital heart disease, cardiomyopathy, and myocarditis, both as a bridge to transplantation and as a bridge to myocardial recovery. The current arsenal of mechanical assist devices available for children is limited to ECMO, intra-aortic balloon counterpulsation, centrifugal pump ventricular assist devices, the DeBakey ventricular assist device Child; the Thoratec ventricular assist device; and the Berlin Heart. In the spring of 2004, five contracts were awarded by the National Heart, Lung and Blood Institute to support preclinical development for a range of pediatric ventricular assist devices and similar circulatory support systems. The support of early development efforts provided by this program is expected to yield several devices that will be ready for clinical trials within the next few years. Our work reviews the current international experience with mechanical circulatory support in children and summarizes our own experience since 2005 with the Berlin Heart, comparing the indications for use, length of support, and outcome between these modalities.

## INTRODUCTION

Most pediatric patients listed for heart transplantation eventually require a high level of cardiac support before transplantation. Currently, over 85% patients undergoing heart transplantation within the Pediatric Heart Transplant Study (PHTS) were United Network of Organ Sharing status 1 (heart failure requiring intravenous inotropes) [1]. Unfortunately, due to the limited availability of donors, only 500 children worldwide undergo cardiac transplantation yearly, and mortality while awaiting a suitable organ exceeds 20% [2]. This situation has led to increased interest in mechanical circulatory support (MCS) as a bridge to transplantation or recovery in the pediatric age group.

Pediatric MCS in the United States has been limited until recently to ECMO, the Bio-pump (Medtronic Bio-Medicus, Minneapolis, Minnesota), and adult systems adapted for pediatric support. New devices in the United States available for children are the implantable MicroMed DeBakey VAD Child (MicroMed Cardiovascular, Inc., Houston, Texas) and the Berlin Heart VAD (Berlin Heart AG, Berlin, Germany) which were granted Humanitarian Device Exemption (HDE), and compassionate use exemption, by the Food and Drug Administration (FDA), respectively. The purpose of this review is to discuss the use of MCS in the critically ill child awaiting heart transplantation, and describe our own experience with the Berlin Heart VAD.

## CURRENT STATE OF THE ART

Implantation of MCS devices in children as a bridge to heart transplantation is not uncommon [3-7]. Both ECMO and VADs have been used successfully in children with cardiorespiratory failure. ECMO initially emerged as a support tool for children in respiratory failure, and subsequently has become the dominant modality of MCS in children. It is generally used for emergencies and short term support while VADs are better utilized for longer term support, mainly as a bridge to transplantation. In instances when recovery seems unlikely and transplantation is the eventful therapy, there is no advantage to employing the ECMO first if VAD technology is available. Based on our experience and the experience of Hetzer *et al.* [8], early application of VAD is crucial to avoid end-organ dysfunction related to inadequate systemic perfusion. A recent report by Blume *et al.* [9] documented favorable outcome in children supported by long term VADs. The report utilized the PHTS database to analyze outcomes in children listed for heart transplantation between 1993 and 2003 placed on VAD support. In the current era (2000-2003), 86% of the VAD supported patients underwent successful heart transplantation. Survival to transplant was significantly poorer in patients with congenital heart disease and in smaller, younger patients. This report demonstrated that postransplantation survival for VAD supported children was not significantly different from that of other status 1 patients who did not require MCS and was significantly superior to Status 1 patients supported with ECMO.

## SHORT-TERM MCS

### 1. ECMO

ECMO is still the most common type of MCS used in infants and children. Indications for ECMO include the pre and postoperative support in congenital heart disease, as well as cardiogenic shock associated with cardiomyopathies, acute myocarditis, and intractable arrhythmias [10]. In patients with congenital heart disease hypoxia and pulmonary hypertension are the most common clinical conditions leading to ECMO support prior to surgery [10]. Failure to wean from cardiopulmonary bypass, cardiogenic shock associated with low cardiac output, and cardiac arrest in the immediate postoperative period, is the most common postoperative indications for ECMO [11]. While ECMO is an effective rescue device, it is generally limited to short-term use (less than a month), requires the patient’s immobilization, precludes rehabilitation, and is associated with multiple potential complications including major bleeding and neurological sequelae. Based on the 2006 ECLS registry report, the mortality rate in pediatric patients supported by ECMO was approximately 60% [12]. This rate has been consistent over the last two decades.

### 2. BioMedicus Centrifugal Pump (BCP)

The BCP (Medtronic BioMedicus, Eden Prairie, MN, USA) is a constant speed, nonpulsatile device which can be used in children of all ages. Compared with roller pumps, there is less hemolysis and less significant inflammatory response [13]. A flow probe is necessary with the centrifugal pump due to its afterload dependency, and the inadequate correlation between the set pump speed in RPM and the actual flow generated. Pump speed requires manual adjustments when venous return decreases. It does not include an oxygenator and, therefore, MCS of children with biventricular failure requires the use of two pump heads. This setup is possible although technically cumbersome in a neonate or infant and, therefore, in these patients, ECMO is preferred.

The use of a single BCP in the setting of postoperative cardiac arrest is suboptimal since severe biventricular failure in these children is expected. Intaoperative placement of this type of a centrifugal pump is more common for patients who cannot be weaned from CPB due to isolated left ventricular failure, such as in children with anomalous left coronary artery arising from the pulmonary artery, transposition of the great arteries following arterial switch, and donor heart dysfunction following transplantation [14]. When global biventricular failure is present, either biventricular BCP or ECMO support will be required. In special cases, unloading of the left ventricle by a left BCP will result in improvement in right ventricular filling, as well as improvement in tricuspid valve function [15] eliminating the need for biventricular support.

The operating room provides a controlled environment which allows assessment of BCP versus ECMO support. In this setting, a venous cannula is placed in the left atrium while clamping the right atrial or caval cannulae used for CPB. Right heart hemodynamic profile is monitored at 150 ml/kg/min pump flow. If right atrial pressure, pulmonary artery pressure, and right ventricular function are satisfactory, the patient is placed on full ventilatory support, while gas exchange in the oxygenator is temporarily interrupted. If gas exchange, metabolic balance, and hemodynamic profile are adequate, left BCP may be utilized. ECMO or biventricular BCP are required if right sided failure is observed during this assessment [16]. Advantages of left BCP over ECMO in those children with pure LV failure is its relative ease of use, fast set-up time, low priming volume, and low-level anticoagulation.

Both modalities are limited to similar short-term periods of support, which is largely responsible for the nearly identical hospital outcomes reported in 1999 by Duncan *et al*. [17] in a single center experience comparing BCP and ECMO use in children. In this report, approximately two thirds of patients supported with either modality were successfully weaned, and 40% in each group survived to hospital discharge. Long term follow-up of the same cohort of patients was reported [16]. The surviving children who required ECMO or BCP support demonstrated favorable long-term survival, overall general health, and cardiac outcomes. Poor neurological outcomes were more common in ECMO than in BCP-supported patients. More than 60% of the ECMO supported patients demonstrated moderate to severe neurological impairment, compared to 20% of the VAD survivors. This advantage may be related to the lower level of anti-coagulation required for VAD support, although the difference may also be related to the use of ECMO in instances of cardiac arrest and low cardiac output which might be expected to substantially increase the risk for neurological complications in neonates and infants.

### 3. Intra-Aortic Balloon Counterpulsation (IABP)

One of the most common MCS devices in adults with left ventricular failure associated with ischemic heart disease is IABP. IABP uses the concepts of systolic unloading and diastolic augmentation. Its use in children remains limited due to technical considerations related to small patient and blood vessel size which complicates insertion and renders the IABP less effective. Early concerns about whether effective counterpulsation was achievable in the highly elastic and distensible aorta of young children have proven unfounded [18]. 

Its clinical efficacy in adults with left ventricular failure was established in 1973 through a multicenter trial [19]. The first experience in children was reported by Pollock and his group from Toronto [20]. They successfully used the IABP in 6 of 14 children following cardiac surgery. A second single center study in 8 children (ages 6 weeks to 6 years old) supported by IABP postoperatively was performed by Veasy and his group in Salt Lake City between November 1981 and November 1982 [21]. Four of the 8 were successfully placed on IABP, but 2 died after 5 and 10 days of support. The two youngest and smallest patients were the only long-term survivors. A more recent work describes the Royal Liverpool Children’s Hospital, postoperative IABP experience in children. 14 patients aged 7 days to 13 years were supported. Ten of the 14 patients (71%) were successfully weaned from the IABP, of whom 8 became long-term survivors (57%) [22]. A significant concern in children with postoperative cardiogenic shock is its association with biventricular failure rather than pure LV failure which is more common in adults with ischemic heart disease. Biventricular support which cannot be achieved with IABP, leads to its limited use in pediatric patients.

## LONG TERM MCS

### 1. Adult Systems Used for Pediatric Support-Heartmate VAD and ThoratecVAD (Thoratec Corp., Pleasanton, CA)

These adult FDA approved extracorporeal MCS devices have been used successfully in older children whose body surface area is greater than 1.4 m^2^ [23-26]. A retrospective multicenter study conducted by Reinhartz and colleagues [25] in children and adolescents supported with the Thoratec VAD (November 1982 until December 1999) revealed overall favorable outcomes. 58 patients in 27 centers worldwide were studied. Their mean age was 13.8 years (range, 7-17 years), and mean weight and body surface area were 51.6 kg (range, 17 to 93 kg) and 1.5 m^2 ^(range, 0.7-2.1 m^2^), respectively. Overall survival in this cohort (71%) was similar to survival in adults supported by this device (58%-71.6%) with a comparable average duration of support. Statistical analysis exposed congenital heart disease as a strong independent risk factor for death. Another concerning finding was that 27% of the subjects had neurologic complications, substantially higher than the 5-12% neurological complications in adults supported by the Thoratec VAD.

### 2. The Micromed DeBakey VAD Child (Micromed Technology Inc., Houston TX, USA)

The DeBakey VAD *Child *system consists of four subsystems: an implantable pump system, an external controller system, an external Clinical Data Acquisition System and an external Patient Home Support System. The blood pump, intended to provide mechanical assistance to the failing left ventricle, is a miniaturized, implantable, titanium, electromagnetically actuated axial flow pump. The pump is 1.2 inches (30.5 mm) in diameter, 3.0 inches (76.2 mm) in length and weighs 95 grams. A titanium inflow cannula connects the pump to the ventricular apex and a Vascutek Gelweave vascular graft (outflow conduit) connects the pump to the aorta. Blood flow from the pump is measured by an ultrasonic flow probe placed around the outflow conduit. The flow probe's wiring is bundled with the pump motor's wiring in a polymer-coated assembly. The cable assembly exits the skin superior to the iliac crest on the right frontal portion of the body and attaches to the VAD's external controller system.

The DeBakey VAD Child was authorized by the U.S. FDA (February 2004) for use in providing temporary left side mechanical circulatory support as a bridge to heart transplantation for pediatric patients (5-16 yeas old, with BSA > 0.7 m^2^ and < 1.5 m^2^**) **who are in NYHA Class IV end stage heart failure, are refractory to medical therapy, and who are listed for heart transplantation. Its use in children has been limited to date. A recent report by Fraser *et al*. [27] summarized a single center experience of 6 patients with the DeBakey VAD *Child*. The average age of the patients was 11 years (range, 6 to 15 years) with a BSA of 0.8 to 1.7 m^2^. The average duration of support was 39 days, with 84 days being the longest duration of support. Three of these patients were successfully transplanted, whereas three died during support before transplantation.

### 3. Pneumatic Pulsatile Pediatric VADs-The Berlin Heart EXCOR and Medos HIA

**Fig. (1):**
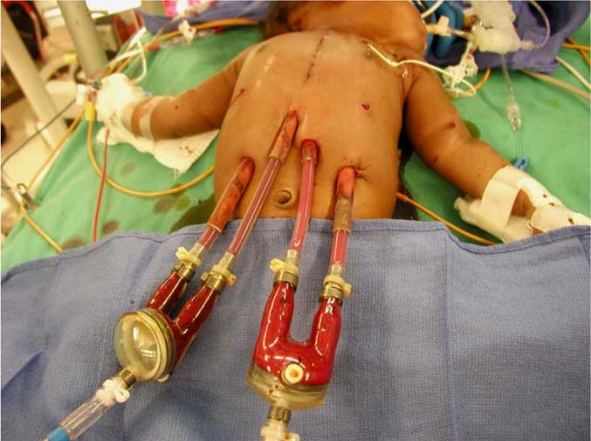
This picture illustrates the RVAD and LVAD cannulae. The order of the cannulae, from right to left, is right atrial outflow, pulmonary artery inflow, aortic inflow and left ventricular outflow.

These extracorporeal systems have provided successful MCS in children of all ages (Fig. **1**). They provide a long term bridge to heart transplantation while allowing extubation, ambulation, and active physical therapy, unlike ECMO or centrifugal pump VADs. While the Berlin Heart EXCOR system has been used successfully in Europe since 1991, the U.S. FDA has only recently approved the use of the Berlin Heart EXCOR Pediatric VAD under a limited investigational device exemption in the USA. 

**Fig. (2):**
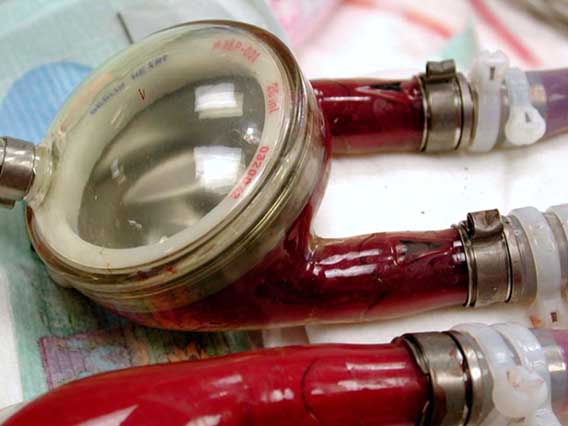
The Berlin Heart VAD (Berlin Heart AG, Berlin, Germany) consists of a paracorporeal, pneumatically driven pump. The pump is made of a translucent, semirigid housing of polyurethane divided into a blood chamber and an air chamber by a three layer flexible diaphragm.

**Fig. (3):**
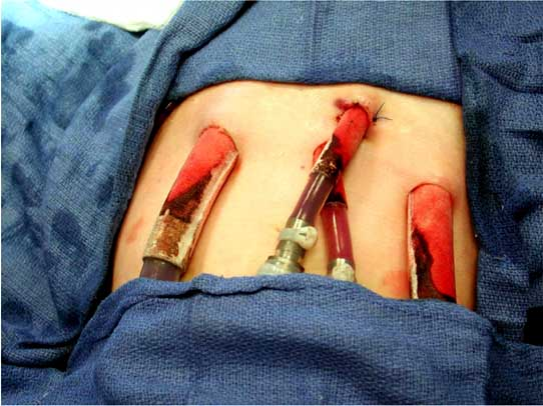
The Berlin Heart VAD cannulae are designed in various configurations to allow biventricular support for all age groups. They are made of silicone rubber with a smooth internal surface. As demonstrated in the exit sites in this figure, the outside of the cannulae is covered with a Dacron (C. R. Bard, Haverhill, Pennsylvania) velour surface at the contact site with the abdominal wall to encourage scar tissue ingrowth, thereby minimize ascending infections.

The Berlin Heart VAD (Berlin Heart AG, Berlin, Germany) consists of a paracorporeal, pneumatically driven pump (Fig. **2**). The pump is made of a translucent, semirigid housing of polyurethane divided into a blood chamber and an air chamber by a three layer flexible diaphragm. Available pump stroke volumes are 10, 25, 30, 50, 60, and 80 mL. The blood flow is directed by trifleaflet polyurethane valves mounted in the pediatric pumps (10, 25, 30 mL), whereas either tilting disc valves (Sorin Biomedical, Torino, Italy) or trileaflet polyurethane valves are available for the adult pump sizes (50, 60, 80 mL). All blood-contacting surfaces inside the pump, including the polyurethane valves, are coated with heparin by the Carmeda process (Carmeda, Upplands Väsby, Sweden). Cannulae are designed in various configurations to allow biventricular support for all age groups. They are made of silicone rubber with a smooth internal surface. The outside of the cannulae is covered with a Dacron (C. R. Bard, Haverhill, Pennsylvania) velour surface at the contact site with the abdominal wall to encourage scar tissue ingrowth, thereby minimize ascending infections (Fig. **3**). The cannulae are designed to produce a considerably reduced afterload to the native heart in comparison with conventional CPB cannulae. All Berlin Heart EXCOR cannulae are made to exit the body through the upper abdominal wall. Inflow cannulation for the left ventricular assist device is typically completed via the left ventricular apex and outflow cannulation is to the ascending aorta. Right atrial to main pulmonary artery cannulation is the preferred method for right-sided support. The driving subunits apply negative pressure to facilitate inflow and positive pressure to drive outflow. There are three compressor subunits. Two are driving the right and left pumps and the third is a backup unit which will take over in case of a subunit failure. The backup unit is designed to allow adequate biventricular positive/negative pressures at a constant rate of 90 per minute should both subunits malfunction simultaneously. Manual operation of the pump is possible in case of complete electrical failure. The maximum positive driving pressure is 350 mm Hg, and the maximum negative driving pressure is minus 100 mm Hg. The available biventricular operating modes are synchronous, where both ventricles are filled and emptied in concert, asynchronous, where one ventricle is filled while the other is emptied, and separate, where each ventricle is cycling independently [28].

A recent retrospective analysis performed by Hetzer and associates in Berlin [8] compared outcomes of children who were supported by the Berlin Heart EXCOR between 1991 and 1998 (period 1; n=34), and children supported by this device between 1999 and 2004 (period 2; n=28). The primary outcomes were survival (defined as 30-day survival, heart transplantation, or myocardial recovery) and hospital discharge, namely, discharge home or to a rehabilitation center. Discharge from the hospital after either weaning from the system or heart transplantation was achieved for 35% in period 1 and for 68% in period 2 (*p*=0.029). Whereas in period 1 there were no survivors in the group of children younger than 1 year old, during period 2, survival in this age group was similar to that of the two groups of older children (*p *=0.024). There was a significant improvement in the discharge rate in period 2 in patients with cardiomyopathy (43% versus 76%, *p *= 0.045) and postcardiotomy heart failure (0% versus 57%, *p=* 0.01). The significant difference in outcomes is reflected in the experience accumulating worldwide in MCS for children.

The first child implanted with the Berlin Heart EXCOR in North America (University of Arizona College of Medicine, Tucson, Arizona) was a 9 year old child with tricuspid insufficiency [29]. Over 160 have now been supported by this device in North America through September 2008 [2]. The device has been used in over 30 US medical centers. Malaisrie *et al.* at Stanford, California have described their experience in 8 children, aged 4 to 55 months [30]. All patients were in cardiogenic shock requiring multiple inotropes. Five patients (63%) were successfully bridged to transplantation; of these, 4 were discharged home and 1 died from early graft failure. Five patients developed post-operative neurologic events. Device exchange was performed in 4 patients in the intensive care unit.

We have implanted Berlin Heart BiVADs in 13 patients from April 2005 to August 2008 [31] The median age of the patients was 2 years (12 days to 17 years). The median patient weight was 10.3 kg (3 to 45 kg). The etiology of heart failure was cardiomyopathy in 11 children and complex congenital heart disease in 2. All children were supported with multiple intravenous inotropes ± mechanical ventilation (9 patients) or ECMO (4 patients) prior to BiVAD implantation. All had severe systemic ventricular dysfunction. At least moderate right ventricular dysfunction, as defined by a 2-dimensional echocardiographic ejection fraction less than 40%, was present in all patients. The median ventilatory requirement post VAD insertion was 4 days (1-22 days). The median length of stay in the intensive care unit was 8.8 days (1-65 days). The median duration of VAD support was 33 days (1-77 days). Twelve of the 13 patients (92%) who received VADs survived the period of circulatory support. A 3 kg baby who required immediate ECMO following birth died from anuric renal failure post Berlin Heart insertion. Three patients had 4 infectious complications. There was one episode of postoperative renal insufficiency not requiring dialysis. There were 18 total pump changes secondary to fibrin deposition or thrombus in 8 patients. One patient suffered a hemidiaphragm paralysis necessitating plication. There was an acute mechanical failure of the IKUS driver in one instance, necessitating manual pumping until the substitute driver was attached. One patient required mediastinal reexploration in the very early postoperative period related to bleeding. There have been no acute neurologic complications and no thromboembolic events. Overall, 12 patients have thus far been successfully bridged to heart transplant. Post-transplant survival has been 100%, with a median follow-up of 24 months. Postoperative anticoagulation is initiated with continuous infusion of heparin 24 hours after admission to the intensive care unit. Partial thromboplastin time is maintained between 40 and 51 seconds during the first 72 hours and 52 to 80 seconds thereafter. Chronic anticoagulation has included: 1. subcutaneous low molecular weight heparin initiated at 1 mg/kg/dose twice daily and the dose is titrated to maintain the anti factor XA level between 0.75 and 1.0 units per milliliter, 2. Aspirin, 5 mg/kg/dose twice daily, and 3. Dipyridamole, 1 mg/kg/dose four times per day.

## ISSUES TO BE RESOLVED

### 1. Single Versus Biventricular Support

Our experience, albeit limited, demonstrates the feasibility of routine use of biventricular (BiVAD) support in pediatric patients. Children, unlike adults, tend to have significant biventricular involvement as well as increased pulmonary vascular resistance associated with severe decompensated heart failure. The pediatric sized hardware facilitated the implantation, and other than slightly longer operative times, we have not encountered significant disadvantages to the addition of the right VAD. We propose that BiVAD support simplifies postoperative management by elimination of the adverse pathophysiological consequences of right ventricular dysfunction. Another significant benefit is improved liver synthetic function contributing to better control of postoperative coagulopathy as well as intravascular oncotic pressure. Adequate oncotic pressure may promote optimization of extravascular lung water as well as better renal perfusion and diuresis. These beneficial physiological consequences may facilitate earlier extubation and therefore more expeditious recovery.

Criteria to guide decision making in regards to the need for biventricular support are not clear-cut. Studies performed by Farrar *et al.* [33], and Kormos *et al.* [34] in adults concluded that the need for biventricular support is more dependent on the adult patient’s clinical status than on hemodynamic parameters. Unfortunately, there are no clear criteria to guide decision making in regards to the need for biventricular support in children with AHFS. These indicators include impaired mental status, fever in the absence of infection, creatinine level and mixed venous oxygen saturation. Another notable variable was the increased need for blood transfusions in patients eventually requiring biventricular support. This was caused by diffuse bleeding unrelated to obvious surgical problems while supported with LVAD alone.

### 2. MCS as a Bridge to Ventricular Recovery

Current studies support the use of MCS as a bridge to transplantation [8]. Both in adults and children the frequency of myocardial recovery in patients supported by long term mechanical assist devices is low. According to the Berlin Heart Institute’s results for the Berlin Heart Excor, between January 1990 and June 2006, 11 children out of 74 were weaned off the device. In the era between October 2000 and June 2006, 8 out of 34 children were weaned and 18 underwent heart transplantation [35]. No child died after weaning. Of those who were weaned in the new era, one had myocarditis, 2 had cardiomyopathy, and 5 congenital heart disease. Out of the 154 adult patients supported by long term VADs at the University of Pittsburgh [36], 6.5% recovered ventricular function sufficient for device removal. Eighty percent of the patients bridged to recovery carried the diagnoses of acute myocarditis or post-partum cardiomyopathy. These results were consistent with those of a previous multicenter study published by Farrar *et al.* in 2002 [37]. With increased availability of the Berlin Heart in North America and Europe, further insight into the clinical scenarios where VADs can be used for myocardial recovery in pediatric heart failure should be forthcoming in the near future.

### 3. Neurological Complications

According to the experience of Hetzer *et al.* [8] which was summarized above, 2 of 28 children supported by the modified system between 1999 and 2004 suffered cerebral strokes. Our patients [31] had no neurological complications, however, since our anticoagulation regime described above is not significantly different from that described by Hetzer *et al* [8], and the number of patients supported by the Berlin-heart VAD in our center is smaller, index of suspicion for cerebral strokes in our future patients shall remain high.

### 4. Sensitization to Human Leukocyte Antigens (HLAs) 

Patients with implantable VAD are at risk for developing antibodies to HLA during support. HLA sensitization is defined as didithiotreitol-treated T-cell panel-reactive antibody (PRA) titer greater than 10% immediately before transplantation. Data presented by McKenna *et al.* [38] suggest that HLA alloimunization may be due to extensive blood transfusion. This might be related to HLA found on leukocytes within the transfusions. According to Kumpati *et al.* [39], alloimunization during VAD support in adults is not constant but increases rapidly at implantation and then decreases. In this study, device type was not a predictor of sensitization. The presence of HLA antibodies can delay transplantation, as a negative prospective crossmatch is required, and require, per our protocol, pretransplantation administration of intravenous immunoglobulin G and cyclophosphamide, as well as peritransplantation plasmapheresis and cyclophosphamide. In our Berlin-heart VAD supported patients, HLA sensitization occurred in 3 patients. All were supported longer than 30 days and all developed extremely elevated (>90%) PRA by enzyme-linked immunosorbent assay. None had positive donor-specific T or B cell retrospective crossmatch. In our patients there has been one episode of rejection with hemodynamic compromise [31].

### 5. Combination of BiVAD Support and Medical Pulmonary Vasodilator Therapy in Children with “Fixed” Pulmonary Hypertension 

Based on our experience [40],even children with extreme pulmonary hypertension related to congenital heart disease may be successful candidates for a heart transplant alone, instead of heart-lung transplant, if treated with BiVAD combined with medical pulmonary vasodilator therapy. In 2 patients with pulmonary vascular resistance index (Rpi)> 10WU x m^2^ unresponsive to pulmonary vasodilator therapy alone, Rpi decreased to 1.4 and 4.6 WU x m^2^, after 33 and 41 days of support, respectively. Pulmonary vascular resistance remained normal without pulmonary vasodilators in these patients within 3 months after heart transplant.

## THE FUTURE

The need for pediatric miniaturized long-term MCS devices fit for use in all age groups including neonates led the U.S. National Heart, Lung, and Blood Institute (NHLBI) to provide funding for their development [32]. The NHLBI is currently supporting five MCS development programs across the country. These 5-years programs were awarded on March 30, 2004 and include the PediaFlow VAD (University of Pittsburgh), The PediPump (The Cleveland Clinic Foundation), Pediatric Cardiopulmonary Assist System (Ension, Inc), The Pediatric Jarvik 2000 (Jarvik Heart), and the Pediatric Ventricular Assist Device (Penn State). The goal of the program is to have these devices ready to begin human trials at the completion of the funding period in 2009. Each one of these devices has unique features, and the expectation is that the 5 devices shall provide solutions to different pediatric clinical conditions.

## References

[1] Naftel DC, Kirklin JK, Hsu DT (2008). Pediatric heart transplantation: 14 years of improving results illustrated by patient specific predictions. J Heart Lung Transplant.

[2] Duncan BW, Burch M, Kirklin JK, Price JF, Canter CE, Kirklin JK (2007). Management of patients awaiting transplantation: Medical, immunologic and mechanical support. Pediatric heart transplantation.

[3] Del Nido PJ, Armitage JM, Ficker FJ (1994). Extracorporeal membrane oxygenation support as a bridge to pediatric heart transplantation. Circulation.

[4] Gajarski RJ, Mosca RS, Ohye RG (2003). Use of extracorporeal life support as a bridge to pediatric cardiac transplantation. J Heart Lung Transplant.

[5] Fiser WP, Yetman AT, Gunselman RJ (2003). Pediatric arteriovenous extracorporeal membrane oxygenation (ECMO) as a bridge to cardiac transplantation. J Heart Lung Transplant.

[6] Helman DN, Addonizio LJ, Marales DLS (2000). Implantable LVADs can successfully bridge adolescent patients to transplant. J Heart Lung Transplant.

[7] Stiller B, Hetzer R, Weng Y (2003). Heart transplantation in children after mechanical circulatory support with pulsatile pneumatic assist device. J Heart Lung Transplant.

[8] Hetzer R, Potapov EV, Stiller B (2006). Improvement in survival after mechanical circulatory support with pneumatic pulsatile ventricular assist devices in pediatric patients. Ann Thorac Surg.

[9] Blume ED, Naftel DC, Bastardi HJ (2006). Outcomes of children bridged to heart transplantation with ventricular assist devices: A multiinstitutional study. Circulation.

[10] Cooper DS, Jacobs JP, Moore L (2007). Cardiac extracorporeal life support: state of the art in 2007. Cardiol Young.

[11] Duncan BW, Duncan BW (2001). Extracorporeal membrane oxygenation for children with cardiac disease. Mechanical support for cardiac and respiratory failure in pediatric patients.

[12] (2006). ECLS Registry Report. Extracorporeal Life Support Organization.

[13] Morgan IS, Codispoti M, Sanger K, Mankad PS (1998). Superiority of centrifugal pump over roller pump in paediatric cardiac surgery: prospective randomised trial. Eur J Cardiothorac Surg.

[14] Karl TR, Horton SB, Duncan BW (2001). Centrifugal pump ventricular assist device in pediatric cardiac surgery. Mechanical support for cardiac and respiratory failure in pediatric patients.

[15] Pavie A, Leger P (1996). Physiology of univentricular versus biventricular support. Ann Thorac Surg.

[16] Ibrahim AE, Duncan BW, Blume ED, Jonas RA (2000). Long-term follow-up of pediatric cardiac patients requiring mechanical circulatory support. Ann Thorac Surg.

[17] Duncan BW, Hraska V, Jonas RA (1999). Mechanical circulatory support in children with cardiac disease. J Thorac Cardiovasc Surg.

[18] Del Nido PJ, Swan PR, Benson LN (1988). Successful use of intraaortic balloon pumping in a 2-kg infant. Ann Thorac Surg.

[19] Scheidt S, Wilner G, Muyeller H (1973). Intra-aortic balloon counterpulsation in cardiogenic shock. Report of a cooperative clinical trial. N Engl J Med.

[20] Pollock J, Charlton MC, Williams WG, Edmond J, Trusler GA (1980). Inraaortic balloon pumping in children. Ann Thorac Surg.

[21] Veasy LG, Blalock RC, Orth JL, Boucek MM (1983). Intra-aortic balloon pumping in infants and children. Circulation.

[22] Akomea-Agyin C, Kejriwal NK, Franks R, Booker PD, Pozzi M (1999). Intraaortic balloon pumping in children. Ann Thorac Surg.

[23] Helman DN, Addonizio LJ, Morales DL (2000). Implantable left ventricular assist devices can successfully bridge adolescent patients to transplant. J Heart Lung Transplant.

[24] McBride LR, Naunheim KS, Fiore AC, Moroney DA, Swartz MT (1999). Clinical experience with 111 thoratec ventricular assist devices. Ann Thorac Surg.

[25] Reinhartz O, Keith FM, El-Banayosy A (2001). Multicenter experience with the thoratec ventricular assist device in children and adolescents. J Heart Lung Transplant.

[26] Potapov EV, Stiller B, Hetzer R (2007). Ventricular assist devices in children: current achievements and future perspectives. Pediatr Transplant.

[27] Fraser CD Jr, Carberry KE, Owens WR (2006). Preliminary experience with the MicroMed DeBakey pediatric ventricular assist device. Semin Thorac Cardiovasc Surg Pediatr Card Surg Annu.

[28] Merkle F, Boettcher W, Stiller B, Hetzer R (2003). Pulsatile mechanical cardiac assistance in pediatric patients with the Berlin heart ventricular assist device. J Extra Corpor Technol.

[29] Arabía FA, Tsau PH, Smith RG (2006). Pediatric bridge to heart transplantation: application of the Berlin Heart, Medos and Thoratec ventricular assist devices. J Heart Lung Transplant.

[30] Malaisrie SC, Pelletier MP, Yun JJ (2008). Pneumatic paracorporeal ventricular assist device in infants and children: initial Stanford experience. J Heart Lung Transplant.

[31] Gandhi SK, Huddleston CB, Balzer DT, Epstein DJ, Boschert TA, Canter CE (2008). Biventricular assist devices as a bridge to heart transplantation in small children. Circulation.

[32] Baldwin JT, Borovetz HS, Duncan BW (2006). The National Heart, Lung, and Blood Institute Pediatric Circulatory Support Program. Circulation.

[33] Farrar DJ, Hill JD, Pennington DG (1997). Preoperative and postoperative comparison of patients with univentricular and biventricular support with the thoratec ventricular assist device as a bridge to cardiac transplantation. J Thorac Cardiovasc Surg.

[34] Kormos RL, Gasior TA, Kawai A (1996). Transplant candidate’s clinical status rather that right ventricular function defines need for univentricular versus biventricular support. J Thorac Cardiovasc Surg.

[35] Potapov EV, Stiller B, anf Hetzer R (2007). Ventricular assist devices in children: Current achievements and future perspectives. Pediatr Transplant.

[36] Simon MA, Kormos RL, Murali S (2005). Myocardial recovery using ventricular assist devices: Prevalence, clinical characteristics, and outcomes. Circulation.

[37] Farrar DJ, Holman WR, McBride LR (2002). Long-term follow-up of Thoratec ventricular assist device bridge-to-recovery patients successfully removed from support after recovery of ventricular function. J Heart Lung Transplant.

[38] McKenna DH Jr, Eastlund T, Segall M, Noreen HJ, Park S (2002). HLA alloimmunization in patients requiring ventricular assist device support. J Heart Lung Transplant.

[39] Kumpati GS, Cook DJ, Blackstone EH (2004). HLA sensitization in ventricular assist device recipients: does type of device make a difference?. J Thorac Cardiovasc Surg.

[40] Gandhi SK, Grady RM, Huddleston CB, Balzer DT, Canter CE (2008). Beyond Berlin: heart transplantation in the "untransplantable". J Thorac Cardiovasc Surg.

